# Plasma neuropeptide Y levels and adverse clinical outcomes after acute ischaemic stroke

**DOI:** 10.1111/ene.16548

**Published:** 2024-11-22

**Authors:** Wenjing Dong, Yaling Lu, Jiayi Long, Yanbo Peng, Zhong Ju, Tan Xu, Yonghong Zhang, Guojie Zhai, Chongke Zhong

**Affiliations:** ^1^ Department of Epidemiology, School of Public Health Jiangsu Key Laboratory of Preventive and Translational Medicine for Geriatric Diseases, MOE Key Laboratory of Geriatric Diseases and Immunology, Suzhou Medical College of Soochow University Suzhou China; ^2^ Department of Neurology Affiliated Hospital of North China University of Science and Technology Tangshan China; ^3^ Department of Neurology Kerqin District First People's Hospital of Tongliao City Tongliao China; ^4^ Department of Neurology Suzhou Ninth People's Hospital, Suzhou Ninth Hospital Affiliated to Soochow University Suzhou China

**Keywords:** biomarker, ischaemic stroke, neuropeptide Y, prognosis, risk prediction

## Abstract

**Background and purpose:**

Neuropeptide Y (NPY) has been reported to be involved in the pathophysiology of several cardiovascular disease processes and might contribute to the incidence of stroke, but the prognostic utility of circulating NPY after acute ischaemic stroke is unclear. This study aimed to prospectively examine the association between plasma NPY levels and adverse clinical outcomes after acute ischaemic stroke.

**Methods:**

Plasma NPY levels were measured in 3250 patients (2066 men and 1184 women) from the China Antihypertensive Trial in Acute Ischaemic Stroke. The primary outcome was the combination of death and major disability (modified Rankin Scale score ≥3) at 12 months after stroke onset, and secondary outcomes included major disability, death and cardiovascular events.

**Results:**

During the 12‐month follow‐up, 702 participants (21.6%) experienced major disability or died. After multivariable adjustment, odds ratio (95% confidence interval) for the highest quartile of NPY was 1.56 (1.19–2.04) for the primary outcome, compared to the lowest quartile. Each standard deviation (0.27 ng/mL) higher log‐transformed NPY was associated with an odds ratio (95% confidence interval) of 1.18 (1.07–1.30) for the primary outcome, 1.28 (1.15–1.42) for major disability. The addition of NPY to a conventional risk factors model improved risk prediction of the composite outcome of death and major disability (category‐free net reclassification index 8.82%, *p* = 0.040; integrated discrimination improvement 0.38%, *p* = 0.011).

**Conclusions:**

Elevated plasma NPY levels in the acute phase of ischaemic stroke were associated with increased risk of poor clinical outcomes after ischaemic stroke, suggesting that plasma NPY may be a potential prognostic biomarker for ischaemic stroke.

## INTRODUCTION

With rising average life expectancy, stroke has become the second leading cause of disability and death worldwide [1]. As established risk factors including age, stroke severity, atrial fibrillation and diabetes mellitus fail to fully explain the adverse clinical outcomes of ischaemic stroke, explorations for novel biomarkers to refine risk prediction and to better understand the pathogenesis of the prognosis of stroke are necessary [2, 3]. Neuropeptide Y (NPY), a 36 amino acid neuropeptide, is abundantly distributed in the mammalian central nervous system and peripheral tissues [4, 5]. Through binding to its receptors, NPY is a crucial player in a variety of physiological functions, including cardiac remodelling, angiogenesis, blood pressure (BP) regulation and neural stem cell proliferation [6, 7]. NPY is directly involved in the pathophysiology of atherosclerosis by affecting the proliferation and migration of vascular smooth muscle cells, the vascular endothelial dysfunction, the formation of foam cells and the aggregation and adhesion of platelets, and is also reported to be involved in the regulation of neurological diseases [7, 8].

The association between circulating NPY and multiple arteriosclerotic cardiovascular disease risk factors, including smoking, obesity, type 2 diabetes mellitus and hypertension, has been revealed [8, 9, 10]. NPY polymorphism was suggested to be a genetic risk factor for the incidence of stroke, suggesting the possible involvement of NPY systems in stroke pathology mechanisms [11, 12, 13, 14]. Moreover, peripheral or central administration of NPY in middle cerebral artery occlusion stroke increased the relative infarct volume and suppressed the regional cerebral blood flow during reperfusion [15]. Experimental study has also found that NPY‐Y1 receptor activation can contribute to nitric oxide overproduction during ischaemic injury, worsening the outcome of cerebral ischaemia [16]. Furthermore, intracerebral haemorrhage patients with a good prognosis were reported to have lower serum NPY levels, suggesting the potential of NPY for predicting prognosis [17]. Several population‐based studies have demonstrated that elevated NPY level is associated with cerebral vasospasm and consecutive stroke due to subarachnoid haemorrhage [18, 19]. However, there is limited research on the relationship between circulating NPY levels and the risk of adverse clinical outcomes after ischaemic stroke.

Based on data derived from the China Antihypertensive Trial in Acute Ischaemic Stroke (CATIS), the purpose of this prospective study is to investigate the association between plasma NPY and death and major disability in acute ischaemic stroke patients, and to examine the prognostic potential of NPY.

## METHODS

### Study design and participants

The CATIS trial was a multicentre, single‐blind, blinded endpoints randomized clinical trial conducted in 26 hospitals across China between August 2009 and May 2013. The detailed design and main results of the trial have been described previously [20]. In brief, a total of 4071 patients aged ≥22 years who had first‐ever ischaemic stroke confirmed by computed tomography or magnetic resonance imaging of the brain within 48 h of symptom onset and elevated systolic BP between 140 and 220 mmHg were recruited. The exclusion criteria of CATIS were as follows: (i) systolic BP ≥220 or diastolic BP ≥120 mmHg; (ii) treated with intravenous thrombolytic therapy; (iii) having severe heart failure, acute myocardial infarction, unstable angina, atrial fibrillation, aortic dissection, cerebrovascular stenosis, resistant hypertension or in a deep coma. For the present study, several participants were further excluded because they did not offer blood samples, or some collected samples were haemolysed in storage or transport, or because plasma NPY levels were not determined. Finally, a total of 3250 ischaemic stroke patients were included in this study.

### Standard protocol approvals, registrations and patient consents

The CATIS protocol was approved by the Institutional Review Boards or Ethical Committees at Soochow University in China and Tulane University in the United States, and all participating hospitals. Written consents were obtained from all study participants or their immediate family members. The CATIS trial was registered at clinicaltrials.gov (NCT01840072).

### Data collection

Fasting blood samples were drawn within 24 h of patients' hospital admission. Plasma samples were separated at clinical laboratories of each participating hospital and immediately frozen at −80°C until laboratory testing. Plasma NPY concentrations were measured centrally in the Central Laboratory of the School of Public Health at Soochow University with a commercially available enzyme‐linked immunosorbent assay (ELISA) kit (R&D Systems, Minneapolis, MN, USA). Intra‐assay and inter‐assay coefficients of variation were 3.0% and 5.2%, respectively. Laboratory technicians who measured plasma NPY concentrations were blind to the baseline characteristics and clinical outcomes of patients.

Baseline data on demographic characteristics, clinical features, medical history and medication use history were collected at the time of enrolment using a standard questionnaire. The definition of medical history was history of hypertension (patient's BP ≥140/90 mmHg on repeated measurements or on antihypertensive medication before stroke onset), diabetes mellitus (fasting blood glucose level ≥120 mg/dL or use of antidiabetic drugs before stroke onset) or dyslipidaemia (total cholesterol measurement ≥240 mg/dL, high‐density lipoprotein measurement <35 mg/dL or use of lipid‐lowering agents before stroke onset) [21]. Stroke severity was assessed with the National Institutes of Health Stroke Scale (NIHSS) by trained neurologists at baseline [22]. Three BP measurements were obtained at admission by trained nurses using a standard mercury sphygmomanometer according to a standard protocol adapted from procedures recommended by the American Heart Association [23]. In addition, serum lipids, plasma glucose and other clinical laboratory measurements were performed on fresh blood samples at each participating hospital at admission.

### Study outcomes

Participants were followed up in person at 12 months after ischaemic stroke by trained neurologists unaware of treatment assignment. The primary outcome was a combination of death and major disability (modified Rankin Scale [mRS] score of 3–6). Scores on the mRS range from 0 to 6, with a score of 0 indicating no symptoms, a score of 5 indicating severe disability (i.e., bedridden, incontinent, or requiring constant nursing care and attention) and a score of 6 indicating death. Secondary outcomes included major disability (mRS score 3–5), death and cardiovascular events (e.g., vascular deaths, nonfatal stroke, nonfatal myocardial infarction, hospitalized and treated angina, hospitalized and treated congestive heart failure, and hospitalized and treated peripheral arterial disease). Death certificates were obtained for deceased participants, and hospital data were abstracted for all cardiovascular events. The study outcomes assessment committee, unaware of treatment assignment, reviewed and adjudicated subsequent outcomes using the criteria established in the Antihypertensive and Lipid‐lowering Treatment to Prevent Heart Attack Trial.

### Statistical analysis

All study participants were divided into four groups according to quartiles of plasma NPY concentrations at admission, and baseline characteristics were compared amongst quartiles. Tests for linear trend of baseline characteristics across NPY quartiles were performed using generalized linear regression analysis for continuous variables and Cochran–Armitage trend *χ* ^2^ test for categorical variables.

Logistic regression and Cox proportional hazards models were performed to estimate the relationships between plasma NPY levels and study outcomes. Odds ratios (ORs), hazard ratios and 95% confidence intervals (CIs) were calculated for higher quartiles compared with the lowest quartile and for a one standard deviation (SD) increment of logarithm‐transformed NPY (0.27 pg/mL). Potential covariates for adverse clinical outcomes of ischaemic stroke were selected based on prior literature. The covariates included in the multivariable models were age, sex, current smoking, alcohol drinking, body mass index, time from onset to randomization, admission NIHSS score, systolic BP, total cholesterol, uric acid, high‐sensitivity C‐reactive protein, hypertension, coronary heart disease, diabetes mellitus, family history of stroke and ischaemic stroke subtype. In addition, spline regression models were used to explore the shapes of associations between plasma NPY levels and study outcomes, fitting a restricted cubic spline function with four knots (at the 5th, 35th, 65th and 95th percentiles) [24].

Because previous studies have reported that NPY levels were associated with several stroke‐related risk factors (i.e., hypertension, obesity and diabetes mellitus), subgroup analyses were further conducted to assess the potential effect modification on the association of plasma NPY with the primary outcome by these covariables [10, 25, 26]. Interactions between NPY levels and subgroup variables on the primary outcome were tested by the likelihood ratio test of models with interaction terms, adjusting for the aforementioned covariates. Moreover, the *C* statistic, net reclassification index (NRI) and integrated discrimination improvement (IDI) were calculated to evaluate the incremental predictive value of plasma NPY beyond conventional risk factors [27]. The conventional model included age, sex, current smoking, alcohol drinking, body mass index, time from onset to randomization, admission NIHSS score, systolic BP, total cholesterol, uric acid, high‐sensitivity C‐reactive protein, hypertension, coronary heart disease, diabetes mellitus, family history of stroke and ischaemic stroke subtype. Multiple imputation for missing data was performed using the Markov chain Monte Carlo method. All *p* values were two‐tailed, and *p* < 0.05 was considered to be statistically significant. Statistical analyses were conducted with SAS statistical software (version 9.4; SAS Institute, Cary, NC, USA).

## RESULTS

### Baseline characteristics

A total of 3250 patients (2066 men and 1184 women) with a mean age (SD) of 61.7 (10.8) years were analysed in our study. The median plasma NPY concentration was 3785.75 pg/mL (interquartile range 2533.07–5551.93 pg/mL) and the median time from symptom onset to admission was 10.0 h (interquartile range 5.0–24.0 h). Compared to patients with the lowest quartile of plasma NPY levels, those with higher NPY levels were more likely to be younger and female; have lower admission NIHSS score; have higher total cholesterol levels and uric acid levels; have lower prevalence of alcohol drinking, thrombotic and embolic stroke; and have higher prevalence of lacunar stroke. Baseline characteristics of participants according to NPY categories are shown in Table 1.

**TABLE 1:** Baseline characteristics of participants according to quartiles of plasma NPY.

Characteristics^a^	NPY, pg/mL				*p* trend
	<2533.07	2533.07–3785.75	3785.75–5551.93	≥5551.93	
Patients, *n*	813	812	812	813	
Demographics					
Age, years	62.20 ± 10.44	62.71 ± 10.70	61.32 ± 11.07	60.49 ± 10.89	<0.001
Male sex, *n* (%)	560 (68.88)	515 (63.42)	510 (62.81)	481 (59.16)	<0.001
Current cigarette smoking, *n* (%)	288 (35.42)	314 (38.67)	299 (36.82)	275 (33.83)	0.378
Current alcohol drinking, *n* (%)	286 (35.18)	231 (28.45)	260 (32.02)	233 (28.66)	0.028
Clinical features					
Admission systolic BP, mmHg	165.70 ± 16.56	167.21 ± 17.16	165.53 ± 16.89	166.33 ± 16.60	0.932
Admission diastolic BP, mmHg	96.42 ± 11.31	96.74 ± 11.34	96.69 ± 10.69	96.67 ± 10.93	0.699
Body mass index, kg/m^2^	24.93 ± 2.96	24.82 ± 3.21	25.04 ± 3.16	25.09 ± 3.16	0.149
Admission NIHSS score	5.00 (3.00–8.00)	4.50 (3.00–8.00)	4.00 (2.00–7.00)	4.00 (2.00–8.00)	0.033
Triglyceride, mmol/L	1.38 (0.97–2.01)	1.46 (1.02–2.14)	1.66 (1.11–2.47)	1.53 (1.06–2.30)	0.059
Total cholesterol, mmol/L	4.92 (4.20–5.67)	5.00 (4.24–5.75)	5.12 (4.40–5.80)	5.06 (4.33–5.72)	0.021
LDL‐cholesterol, mmol/L	2.88 (2.27–3.48)	2.80 (2.23–3.43)	2.96 (2.30–3.57)	2.91 (2.37–3.47)	0.117
HDL‐cholesterol, mmol/L	1.25 (1.04–1.51)	1.23 (1.04–1.50)	1.22 (1.03–1.49)	1.22 (1.01–1.50)	0.258
Creatinine, μmol/L	69.00 (59.00–81.60)	69.00 (58.25–81.90)	68.00 (56.00–81.00)	70.00 (58.00–83.00)	0.560
Uric acid, μmol/L	280.00 (225.10–339.00)	289.50 (233.04–355.70)	288.45 (235.00–355.90)	290.00 (233.40–361.00)	0.003
High‐sensitivity C‐reactive protein, mg/L	2.00 (0.80–6.30)	2.20 (0.80–5.89)	1.90 (0.70–5.23)	2.00 (0.80–5.00)	0.524
Time from onset to randomization, h	10.00 (5.00–24.00)	10.00 (5.00–24.00)	11.00 (5.00–24.00)	10.00 (4.00–24.00)	0.431
Medical history, *n* (%)					
Hypertension	640 (78.72)	647 (79.68)	619 (76.23)	655 (80.57)	0.744
Hyperlipidaemia	56 (6.89)	56 (6.90)	60 (7.39)	60 (7.38)	0.626
Diabetes mellitus	134 (16.48)	138 (17.00)	163 (20.07)	140 (17.22)	0.377
Coronary heart disease	82 (10.09)	83 (10.22)	88 (10.84)	87 (10.70)	0.608
Family history of stroke, *n* (%)	132 (16.24)	159 (19.58)	162 (19.95)	157 (19.31)	0.117
Medication use history, *n* (%)					
Antihypertensive medications	416 (51.17)	404 (49.75)	398 (49.01)	392 (48.22)	0.221
Lipid‐lowering medications	30 (3.69)	26 (3.20)	31 (3.82)	27 (3.32)	0.865
Ischaemic stroke subtype, *n* (%)					
Thrombotic	660 (81.18)	653 (80.42)	629 (77.46)	594 (73.06)	<0.001
Embolic	64 (7.87)	34 (4.19)	35 (4.31)	27 (3.32)	<0.001
Lacunar	112 (13.78)	148 (18.23)	164 (20.20)	211 (25.95)	<0.001

*Note*: Categorical variables are expressed as frequency (percent).

Abbreviations: BP, blood pressure; HDL, high‐density lipoprotein; LDL, low‐density lipoprotein; NIHSS, National Institutes of Health Stroke Scale; NPY, neuropeptide Y.

^a^ Continuous variables are expressed as mean ± SD or median (interquartile range).

### Plasma NPY levels and clinical outcomes

**FIGURE 1:**
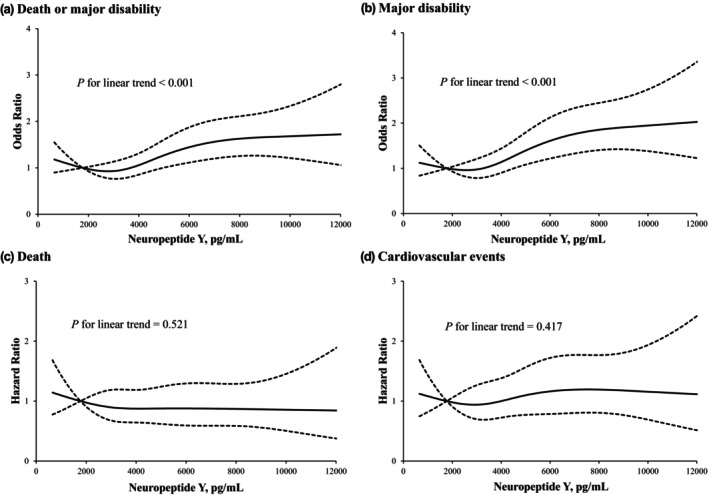
Relationship of plasma NPY with risk of adverse outcome after acute ischaemic stroke. Odds ratios and 95% confidence intervals derived from restricted cubic spline regression, with knots placed at the 5th, 35th, 65th and 95th percentiles of the distribution of plasma NPY. The reference point is the midpoint of the reference group from categorical analysis. Odds ratios were adjusted for age, sex, current smoking, alcohol drinking, body mass index, time from onset to randomization, admission NIHSS score, systolic blood pressure, total cholesterol, uric acid, high‐sensitivity C‐reactive protein, hypertension, coronary heart disease, diabetes mellitus, family history of stroke and ischaemic stroke subtype. (a) Combined outcome, (b) major disability, (c) death and (d) cardiovascular events.

After 12 months of follow‐up, 702 participants (21.6%) experienced major disability or died (509 major disability and 193 deaths), and 186 cardiovascular events occurred. After adjustment for age, sex, admission NIHSS score, medical history, ischaemic stroke subtype and other important prognostic factors, compared with the lowest quartile the ORs (95% CIs) associated with the highest quartile of plasma NPY were 1.56 (1.19–2.04) for the primary outcome and 1.80 (1.35–2.40) for major disability. A one SD increase of log‐transformed NPY was associated with an increased risk of 18% (OR 1.18, 95% CI 1.07–1.30) for the primary outcome and 28% (OR 1.28, 95% CI 1.15–1.42) for major disability (Table 2). Multivariable‐adjusted restricted cubic spline analyses presented linear associations of NPY levels with the primary outcome (*p* for linearity <0.001) and major disability (*p* for linearity <0.001) (Figure 1).

**TABLE 2:** Risk of 12‐month clinical outcomes according to plasma NPY quartiles in the acute phase of ischaemic stroke.

	NPY, pg/mL				*p* trend	Each SD (0.27 pg/mL) increase in logarithm NPY
	<2533.07	2533.07–3785.75	3785.75–5551.93	≥5551.93		
Median, pg/mL	1770.95	3146.71	4540.54	7355.00		
Cases, *n* (%)	176 (22.56)	153 (19.52)	172 (21.83)	201 (25.48)	–	–
Age‐ and sex‐adjusted OR	1.00	0.81 (0.63–1.03)	1.00 (0.78–1.28)	1.29 (1.01–1.64)	0.012	1.10 (1.00–1.20)
Multiple‐adjusted OR^a^	1.00	0.84 (0.64–1.11)	1.13 (0.86–1.49)	1.56 (1.19–2.04)	<0.001	1.18 (1.07–1.30)
Major disability (mRS score 3–5)						
Cases, *n* (%)	119 (14.64)	109 (13.42)	121 (14.90)	160 (19.68)	–	–
Age‐ and sex‐adjusted OR	1.00	0.89 (0.67–1.18)	1.05 (0.80–1.39)	1.54 (1.18–2.01)	<0.001	1.19 (1.08–1.32)
Multiple‐adjusted OR^a^	1.00	0.93 (0.69–1.26)	1.18 (0.88–1.59)	1.80 (1.35–2.40)	<0.001	1.28 (1.15–1.42)
Death						
Cases, *n* (%)	57 (7.01)	44 (5.42)	51 (6.28)	41 (5.04)	–	–
Age‐ and sex‐adjusted HR	1.00	0.73 (0.50–1.09)	0.90 (0.62–1.32)	0.76 (0.51–1.14)	0.334	0.88 (0.77–1.01)
Multiple‐adjusted HR^a^	1.00	0.77 (0.52–1.14)	0.94 (0.64–1.37)	0.82 (0.55–1.23)	0.524	0.91 (0.79–1.04)
Cardiovascular events						
Cases, *n* (%)	52 (6.40)	41 (5.05)	43 (5.30)	50 (6.15)	–	–
Age‐ and sex‐adjusted HR	1.00	0.77 (0.51–1.17)	0.84 (0.56–1.25)	1.00 (0.68–1.48)	0.918	0.99 (0.86–1.15)
Multiple‐adjusted HR^a^	1.00	0.77 (0.51–1.17)	0.86 (0.57–1.29)	1.06 (0.71–1.56)	0.706	1.01 (0.87–1.18)

Abbreviations: HR, hazard ratio; NIHSS, National Institutes of Health Stroke Scale; NPY, neuropeptide Y; mRS, modified Rankin Scale; OR, odds ratio.

^a^ Adjusted for age, sex, current smoking, alcohol drinking, body mass index, time from onset to randomization, admission NIHSS score, systolic blood pressure, total cholesterol, uric acid, high‐sensitivity C‐reactive protein, hypertension, coronary heart disease, diabetes mellitus, family history of stroke and ischaemic stroke subtype.

The potential modified effects of prespecified factors were further examined. Subgroup analyses revealed that higher plasma NPY levels were significantly associated with the primary outcome in most strata of the aforementioned covariates. Furthermore, no significant interaction between plasma NPY and these prespecified factors was observed (all *p* for interaction >0.05) (Table 3).

**TABLE 3:** Subgroup analyses of the association between plasma NPY and the primary outcome in ischaemic stroke patients.

Subgroup	NPY, pg/mL				*p* trend	*p* interaction
	<2533.07	2533.07–3785.75	3785.75–5551.93	≥5551.93		
Stroke onset age, years						
<65	1.00	0.82 (0.55–1.24)	1.09 (0.74–1.62)	1.60 (1.10–2.31)	0.004	0.780
≥65	1.00	0.86 (0.59–1.25)	1.19 (0.81–1.73)	1.43 (0.96–2.13)	0.032	
Sex						
Women	1.00	0.84 (0.52–1.34)	1.62 (1.02–2.58)	1.70 (1.07–2.70)	0.002	0.246
Men	1.00	0.86 (0.61–1.21)	0.90 (0.63–1.27)	1.52 (1.09–2.13)	0.024	
Current cigarette smoking						
No	1.00	0.87 (0.62–1.22)	1.28 (0.92–1.79)	1.53 (1.10–2.13)	0.002	0.683
Yes	1.00	0.77 (0.48–1.24)	0.85 (0.52–1.39)	1.59 (0.99–2.55)	0.059	
Current alcohol drinking						
No	1.00	0.91 (0.66–1.26)	1.35 (0.97–1.87)	1.65 (1.20–2.28)	<0.001	0.246
Yes	1.00	0.70 (0.41–1.21)	0.73 (0.44–1.23)	1.40 (0.85–2.32)	0.295	
Admission NIHSS score						
<4	1.00	0.76 (0.39–1.51)	1.07 (0.57–2.03)	1.46 (0.80–2.66)	0.097	0.098
≥4	1.00	0.85 (0.64–1.14)	1.13 (0.85–1.51)	1.51 (1.13–2.02)	0.001	
Receiving immediate BP reduction						
No	1.00	0.70 (0.47–1.04)	0.88 (0.59–1.32)	1.70 (1.15–2.51)	0.004	0.207
Yes	1.00	0.97 (0.66–1.44)	1.43 (0.98–2.09)	1.46 (1.00–2.13)	0.015	
History of hypertension						
No	1.00	0.73 (0.39–1.39)	1.76 (0.98–3.17)	1.30 (0.70–2.42)	0.085	0.937
Yes	1.00	0.88 (0.65–1.20)	1.01 (0.74–1.39)	1.66 (1.22–2.24)	<0.001	
Antihypertensive medications						
No	1.00	0.64 (0.43–0.96)	1.15 (0.78–1.70)	1.26 (0.86–1.86)	0.039	0.884
Yes	1.00	1.06 (0.72–1.57)	1.11 (0.75–1.64)	1.93 (1.32–2.83)	0.001	

*Note*: Adjusted for age, sex, current smoking, alcohol drinking, body mass index, time from onset to randomization, admission NIHSS score, systolic blood pressure, total cholesterol, uric acid, high‐sensitivity C‐reactive protein, hypertension, coronary heart disease, diabetes mellitus, family history of stroke and ischaemic stroke subtype, except the variable was used as a stratified variable.

Abbreviations: NPY, neuropeptide Y; BP, blood pressure; NIHSS, National Institutes of Health Stroke Scale.

### Incremental prognostic value of plasma NPY

Whether adding NPY to a model consisting of conventional risk factors could improve the risk prediction of adverse clinical outcomes in patients with acute ischaemic stroke was examined. As shown in Table 4, the addition of NPY to the conventional risk factors model significantly improved the risk discrimination and reclassification for the primary outcome (*C* statistic increased from 0.806 to 0.808, *p* = 0.043; category‐free NRI 8.82%, *p* = 0.040; IDI 0.38%, *p* = 0.011) and for major disability (*C* statistic increased from 0.780 to 0.785, *p* = 0.030; category‐free NRI 13.11%, *p* = 0.007; IDI 0.73%, *p* < 0.001).

**TABLE 4:** Reclassification and discrimination statistics for 12‐month clinical outcomes by plasma NPY amongst patients with acute ischaemic stroke.

	Calibration statistic		NRI (category‐free)		IDI	
	*χ* ^2^	*p* value	Estimate (95% CI), %	*p* value	Estimate (95% CI), %	*p* value
Death or major disability (mRS score 3–6)						
Conventional model	0.806 (0.787–0.824)	–	Reference	–	Reference	–
Conventional model + NPY	0.808 (0.790–0.827)	0.043	8.82 (0.43–17.20)	0.040	0.38 (0.09–0.68)	0.011
Major disability (mRS score 3–5)						
Conventional model	0.780 (0.759–0.801)	–	Reference	–	Reference	–
Conventional model + NPY	0.785 (0.764–0.806)	0.030	13.11 (3.68–22.54)	0.007	0.73 (0.33–1.12)	<0.001
Death						
Conventional model	0.776 (0.743–0.810)	–	Reference	–	Reference	–
Conventional model + NPY	0.776 (0.742–0.810)	0.456	4.50 (−10.03–19.03)	0.544	0.06 (−0.01–0.14)	0.098
Cardiovascular events						
Conventional model	0.632 (0.592–0.673)	–	Reference	–	Reference	–
Conventional model + NPY	0.633 (0.593–0.673)	0.599	0.59 (−14.21–15.39)	0.938	0.00 (−0.03–0.03)	0.775

*Note*: Conventional model included age, sex, current smoking, alcohol drinking, body mass index, time from onset to randomization, admission NIHSS score, systolic blood pressure, total cholesterol, uric acid, high‐sensitivity C‐reactive protein, hypertension, coronary heart disease, diabetes mellitus, family history of stroke and ischaemic stroke subtype.

Abbreviations: CI, confidence interval; IDI, integrated discrimination improvement; mRS, modified Rankin Scale; NIHSS, National Institutes of Health Stroke Scale; NPY, neuropeptide Y; NRI, net reclassification index.

## DISCUSSION

In this large prospective cohort of patients with acute ischaemic stroke, it was found that higher plasma NPY concentrations at baseline were independently associated with greater risk of poor clinical outcome of major disability and death at 12 months after ischaemic stroke onset. Subgroup analyses further confirmed these findings. Furthermore, NPY provided improvements in risk discrimination and reclassification for primary outcome and major disability beyond established risk factors, as evidenced by *C* statistics, the NRI and IDI. These findings indicated that plasma NPY could be a potential biomarker for adverse clinical outcomes in acute ischaemic stroke patients.

Epidemiological evidence indicated that circulating NPY was closely related to multiple arteriosclerotic cardiovascular disease risk factors [8, 9, 10]. Researches had found that enhanced NPY was associated with atherosclerotic lesion formation and vulnerability both in humans and in mice [28, 29]. Increased plasma NPY was proved to contribute to post‐exercise ischaemia in coronary artery disease patients after accepting the bicycle exercise test [30]. Population‐based studies found that higher NPY levels were correlated with a greater risk of hospitalization, heart transplantation and ventricular assist device placement in patients with heart failure, and were associated with cardiovascular events and all‐cause death even after adjustment for other prognostic variables [5, 31]. Plasma NPY was also reported to function as an independent predictor of adverse cardiovascular outcomes in an analysis based on 277 patients with end‐stage renal disease [32]. Furthermore, NPY levels in patients with intracerebral and subarachnoid haemorrhage were reported to be positively correlated with the severity of disease, and showed potential for predicting the occurrence of subsequent stroke due to vasospasm [17, 18]. Several genetic reports have suggested that the C‐399T NPY promoter polymorphism is associated with increased risk for ischaemic stroke [11, 12, 13]. Our study further extended the existing evidence and first validated the prognostic role of plasma NPY in patients with acute ischaemic stroke.

Our findings are consistent with earlier experimental evidence, which found that administration of NPY could reduce cerebral blood flow and increase infarct volume. For example, bolus administration of NPY into the internal carotid artery caused a dose‐dependent decrease in the ipsilateral striatal local cerebral blood flow to the site of administration in rats [33]. Moreover, studies also demonstrated that NPY increased around brain lesions in animal models, and that administration of NPY following stroke increased infarct volume and reduced reperfusion, indicating that NPY is an important contributor in stroke biology [15, 34]. However, NPY has been reported to produce a neuroprotective effect via Y5 and especially Y2 receptors in transient focal cerebral ischaemia in rats, as ventricularly injected specific agonists of these receptors can protect against oxygen–glucose deprivation‐induced neuronal cell death, diminish the infarct volume and improve selected gait parameters in the CatWalk behavioural test [35]. NPY seems to play a dual role in brain ischaemia by interacting with different receptors. Abounader et al. revealed that Y1 receptors were exclusively found in the smooth muscle cells in human brain microvascular cells in culture, where they most probably mediated cerebral vasoconstriction [36]. Additionally, Y1 receptors were reported to present on both the endothelial and the periarterial sides, and circulating NPY could directly influence contractility [37]. Therefore, in the acute phase, circulating NPY might mainly exacerbate ischaemia by interacting with receptors on the endothelial side of cerebral vessels whilst protecting neurons by receptors at other sites.

In the present study, the significant association of plasma NPY with clinical outcomes was independent of well‐established risk factors for poor stroke prognosis, such as age, sex, admission NIHSS score, medical history and ischaemic stroke subtype. More importantly, adding plasma NPY to conventional risk factors significantly improved risk prediction for primary outcome and major disability, suggesting that plasma NPY may be an important prognostic marker for risk stratification in acute ischaemic stroke patients. It was also found that patients with higher NPY levels were more likely to have higher prevalence of lacunar stroke. Our findings were consistent with the evidence of a high frequency of NPY polymorphism in stroke patients, especially the small vessel disease subtype, the profound effect of NPY on the small arterioles of the brain, and direct action on vascular endothelial cells [11, 37, 38]. Clinical trials are needed to assess the effect of relief from elevated NPY with some interventions (including neuropeptide Y1 receptor antagonist) in patients with acute ischaemic stroke [39]. This study has important clinical implications for better understanding of the aetiology of ischaemic stroke, and supports NPY as a potential therapeutic target for an important role in the prognostication of ischaemic stroke.

The mechanisms underlying the observed association of NPY with poor outcomes are not fully clear, but several potential pathophysiological processes have been proposed. Under pathological conditions, NPY binding to Y1 receptor on vascular endothelial cells promoted the mitosis of endothelial cells, which played a key role in the process of intima thickening [40, 41]. Zhou et al. found that the binding of NPY with Y1 receptor could promote the synthesis and release of high‐mobility group box 1 protein from macrophages, which was involved in the inflammatory response and may damage the integrity of the endothelial cell barrier [42]. The NPY‐Y1 receptor activation could also increase the relative brain nitric oxide (NO) concentration and excessive NO mediated ischaemic injury [16]. Further experimental and human investigations are warranted to extend our knowledge of the potential biological mechanism of NPY in ischaemic stroke prognosis.

Several limitations deserve discussion. First, our study was observational and based on the CATIS trial, excluding ischaemic stroke patients with BP ≥220/120 mmHg and systolic BP <140 mmHg, severe heart failure, acute myocardial infarction or unstable angina, atrial fibrillation, aortic dissection, cerebrovascular stenosis or treated with intravenous thrombolytic therapy at admission. A selection bias might be a concern. However, the proportion of patients with BP ≥220/120 mmHg or treated with intravenous thrombolytic therapy is low in China [20, 43]. Second, the possibility of residual confounding cannot be fully eliminated in this observational study although multiple potential confounders were adjusted for. Third, data on large vessel occlusion were not collected, which are of great clinical significance. Fourth, plasma NPY was measured only once at baseline, so it was not possible to assess the dynamic variation of NPY levels amongst patients with acute ischaemic stroke or the association between NPY changes and prognosis of acute ischaemic stroke.

In conclusion, elevated plasma NPY levels in the acute phase of ischaemic stroke were associated with increased risk of major disability, death and major disability at 12 months. Plasma NPY may be a potential prognostic biomarker for risk stratification in acute ischaemic stroke patients.

## AUTHOR CONTRIBUTIONS

**Wenjing Dong:** Writing – original draft; visualization; writing – review and editing. **Yaling Lu:** Writing – original draft; visualization; formal analysis. **Jiayi Long:** Data curation. **Yanbo Peng:** Investigation; data curation. **Zhong Ju:** Investigation; data curation. **Tan Xu:** Conceptualization; methodology. **Yonghong Zhang:** Conceptualization; methodology. **Guojie Zhai:** Conceptualization; methodology; supervision; writing – review and editing. **Chongke Zhong:** Conceptualization; funding acquisition; supervision; methodology; writing – review and editing.

## FUNDING INFORMATION

This study was supported by the National Natural Science Foundation of China (grant numbers 82273706 and 82220108001), Interdisciplinary Basic Frontier Innovation Programme of Suzhou Medical College of Soochow University (YXY2302013), the Project of MOE Key Laboratory of Geriatric Diseases and Immunology (no. JYN202406) and a Project of the Priority Academic Programme Development of Jiangsu Higher Education Institutions, China.

## CONFLICT OF INTEREST STATEMENT

The authors declare no conflict of interest.

## Data Availability

Research data are not shared.
